# Selection bias: neighbourhood controls and controls selected from those presenting to a Health Unit in a case control study of efficacy of BCG revaccination

**DOI:** 10.1186/1471-2288-7-11

**Published:** 2007-02-23

**Authors:** Odimariles MS Dantas, Ricardo AA Ximenes, Maria de Fatima PM de Albuquerque, Ulisses R Montarroyos, Wayner V de Souza, Patrícia Varejão, Laura C Rodrigues

**Affiliations:** 1 Department of Mother and Child Health, Federal University of Pernambuco. Recife, Brasil, Bloco A do Hospital das Clínicas. Av. Moraes Rego s/n. Cidade Universitária, Recife, PE. Brasil; 2 Department of Tropical Medicine, Federal University of Pernambuco. Recife, Brasil, Bloco A do Hospital das Clínicas. Av. Moraes Rego s/n. Cidade Universitária. Recife, PE. Brasil; 3 Department of Internal Medicine, State University of Pernambuco. Recife, Brasil, Núcleo de Pós-Gaduação. Rua Arnóbio Marques 310, Campus Universitário. Santo Amaro. Recife, PE, Brasil; 4 Department of Internal Medicine, Federal University of Pernambuco. Recife, Brasil, Bloco A do Hospital das Clínicas. Av. Moraes Rego s/n. Cidade Universitária. Recife, PE. Brasil; 5 Research Center Aggeu Magalhães, Fundação Oswaldo Cruz. Recife, Brasil, Av. Moraes Rego s/n. Cidade Universitária. Recife, PE. Brasil; 6 London School of Hygiene and Tropical Medicine, University of London. London, UK, Department of Epidemiology and Population Health. London School of Hygiene and Tropical Medicine. Room 258b, Keppel Street. London WC1E 7HT UK

## Abstract

**Background:**

In most case control studies the hardest decision is the choice of the control group, as in the ideal control group the proportion exposed is the same as in the population that produced the cases.

**Methods:**

A comparison of two control groups in a case control study of the efficacy of BCG revaccination. One group was selected from subjects presenting to the heath unit the case attended for routine prevention and care; the second group was selected from the neighbourhood of cases. All Health Units from which controls were selected offered BCG revaccination. Efficacy estimated in a randomized control trial of BCG revaccination was used to establish that the neighbourhood control group was the one that gave unbiased results.

**Results:**

The proportion of controls with scars indicating BCG revaccination was higher among the control group selected from Health Unit attenders than among neighbourhood controls. This excess was not removed after control for social variables and history of exposure to tuberculosis, and appears to have resulted from the fact that people attending the Health Unit were more likely to have been revaccinated than neighbourhood controls, although we can not exclude an effect of other unmeasured variables.

**Conclusion:**

In this study, controls selected from people presenting to a Health Unit overrepresented exposure to BCG revaccination. Had the results from the HU attenders control group been accepted this would have resulted in overestimation of vaccine efficacy. When the exposure of interest is offered in a health facility, selection of controls from attenders at the facility may result in over representation of exposure in controls and selection bias.

## Background

Population controls are increasingly being used in case control studies because of the growing awareness of the limitations of hospital controls, first identified by Berkson (1946) [1]. Essentially, because hospital patients are in hospital (and therefore ill), they are likely to have a higher frequency of hazardous exposures than the population in general; if hospital controls are used, it is necessary to exclude from the control groups people hospitalized for diseases caused by the exposure of interest. Other forms of inclusion criteria for cases and controls can also be used. Population controls, in contrast, are more likely to represent accurately the exposure state in the population that produced the cases. Neighbourhood controls are an alternative to population controls: they represent the exposure in the neighbourhood that produced each case, and therefore tend to control for known and unknown confounding factors that clusters in neighbourhoods[2].

The use of population and neighbourhood controls is not without disadvantages. Population controls can be less willing to participate in research than individuals in a health care setting. If the non responders have a different prevalence of the exposure of interest than responders, the lower response rate can lead to bias[3]. The logistics of data collection for population controls is often more difficult.

There is clearly substantial literature comparing hospital controls to population and neighbourhood controls[4-10]. There is however much less evidence on the vulnerability of bias in controls selected among people registered in the same Health Unit. This is a frequently used source of controls – Health Unit(HU) controls- and includes controls from the same GP practice, some health care provider etc [11-13]. HU controls – controls from the Health Unit the case uses for routine health care- are potentially much better than hospital controls because, if all the population is registered somewhere, those registered in the same Health Unit as the case are unlikely to be less healthy that the population that produced the cases. The robustness against bias is maybe less evident if controls are selected from those **attending **the Health Unit, as is the case in the study discussed here, rather than those **registered **in the Health Unit. A potential vulnerability for bias is created when the exposure of interest is related to the Health Unit (for example an intervention offered at the Health Unit) in particular if controls are selected from attenders (not from those registered) and frequency of attending increases the probability of the subject receiving the measure being studied.

The objective of this paper is to examine the degree of bias for estimated vaccine efficacy (VE) using two control groups: neighbourhood controls and Health Unit attenders controls; and explore the extent to which this bias was caused by differences in the population, and how much caused by the fact that the HU control was a user of the HU.

## Methods

BCG is a vaccine routinely given to prevent tuberculosis in the first year of life. The Brazilian government recommended the use of an additional second dose, given to primary school age children. This recommendation was implemented in some Brazilian states before a decision was taken to undertake a RCT of a second dose of BCG. A case control study was conducted (in parallel to the RCT) in one of the Brazilian states that had introduced routine second dose vaccination. Both case control study and RCT aimed to estimate the additional protection against tuberculosis given by a second dose of BCG vaccine. The methods and results of the RCT have been reported [14,15]. The results of the RCT and the case control study were different, and we decided to investigate the reasons for the difference before publishing the case control study. An examination of potential reasons for this difference identified the possibility of selection bias caused by the control group being selected from attenders of the Health Units.

In the case control study, cases were people with tuberculosis newly diagnosed in the tuberculosis control programme; they were recruited in Health Units that offered tuberculosis treatment. Below we present some relevant aspects of the health system structure and of the control of tuberculosis in Brazil. The health system in Brazil is hybrid. Although there is private medicine and insurance, there is also a health system free at the point of use, with Health Units (with and without teams linked to the Family Health Programme, FHP), secondary care in outpatients and district hospitals, and tertiary care on reference hospitals. The FHP is a new program in which teams of health professionals work in the community linked to Health Units; each team is responsible for about 3200 people living in a defined geographical area. Treatment of tuberculosis is made by Family Health Teams or in Health Units on an out-patients basis (only those very unwell are hospitalized). In Recife, where the study was carried out, decentralization of the TB control program was taking place at the time of the study with the progressive transfer of activities from "tuberculosis Health Units" to FHP and their teams. A total of 102 FHT and 26 Health Units are distributed over 6 Health Districts, located in a way to facilitate the access of those living in low income areas. When necessary, patients may be referred to specialists in Policlinics (10), Special Units of Reference (10) and the Centers of Psychosocial Support. Notification is compulsory, treatment is done exclusively by the tuberculosis control programme, and medicines are released for individual cases only, and only after they are notified, all treatment is free. It is possible that some cases escape diagnosis: mild cases, especially if self-healing may never be diagnosed; and some cases are only diagnosed on autopsy examination (frequently on the homeless). However the number of cases missed in this crowded urban area with a hierarchical, free public health system with primary, secondary and tertiary levels, is likely to be too small to bias any estimate of effect, even if missed cases were more likely, or less likely, to have been vaccinated [16,17].

The controls were Health Unit controls, selected from those attending the Health Unit that cases used for routine medical care before their diagnosis of tuberculosis. Two critical aspects are that they were selected from people attending the Health Unit, rather than those registered; and all 61 Health Units from which Health Unit controls were recruited offered BCG vaccination and revaccination as part of their routine. To investigate the possibility of selection bias, a new control group was selected from the neighbourhood of cases using a systematic approach, starting from the address of the case. The published case control study used the new set of controls[14]. This paper investigates the reasons why these two sets of controls gave different vaccine efficacies.

Both sets of controls were matched to cases by year of birth (which in operational guidance was expressed cases and controls had to belong to the same age group at the year the case was recruited, within the age groups 7–9, 10–14 and 15–19 years). So although neighbourhood controls were selected on average two years later than HU controls, both had the same age group as cases at the time the case was recruited. As neighbourhood controls were selected on average two years later, they had two additional years in which they could have received vaccination. The original case control study ascertained number of BCG scars by examination of the upper arm and vaccination card examined when available. Validity of scar as an indication of BCG vaccination (at least for neonatal vaccination) is good in Brazil [18].

For neighbourhood controls with a vaccination card, we investigated whether the BCG vaccine was received in the previous 2 years: no BCG was received in the two years preceding recruitment. Health Unit controls were recruited during the period December 2001–August 2003, and neighbourhood controls from May 2003–February 2005. Half of Health Unit controls were born before March 1988 and half the neighbourhood controls were born before February 1989. Additional information on demographic variables, on potential confounding variables and on aspects of the disease was collected on a questionnaire applied to cases and to the two control groups. The socio-economic variables treated as confounders are used in the census.

Analysis. This analysis firstly established whether the choice of Health Unit controls caused selection bias by comparing the matched, adjusted estimate VE for each control group. Secondly, it investigated any differences in biological and social variables in the two control groups. Thirdly, it explored whether biological or social characteristics were associated with having two BCG scars, separately in the two control groups, by estimating the OR of having two BCG scars. The final step was to investigate if the bias was removed by controlling for the social biological variables. This was done by a conditional logistic regression. In this analysis, the OR measured the likelihood of receiving a second BCG vaccination in HU controls when compared to neighbourhood controls. The aim of the analysis was to observe if adjustment for potential confounders reduced the magnitude of the OR. An adjusted OR of 1 would indicate that all differences between the two control groups was due to differences in the frequency of social and biological characteristics of the two control groups. Analysis was done using Epi Info version 6.04d (CDC, Atlanta, GA, 2001) and STATA8 (version 6.0; 1999; STATA Corporation, Houston, Texas, USA). The study received ethical approval from the ethical committee of the UFPE. All participants gave written informed consent.

## Results

A higher proportion of Health Unit attender controls had two BCG scars at examination and two BCG vaccinations in the vaccination cards than neighbourhood controls (Table 1); as consequence (adjusted) vaccine efficacy was 8% for population controls and 39% for Health Unit controls.

**Table 1:** Presence of 2 BCG scars and two BCG vaccinations in the vaccination card in Health Unit and neighbourhood controls

	**Cases**	**Health Unit Controls**	**Neighbourhood Controls**
**BCG Scar**			
One	75 (44.4%)	180 (32.7%)	213 (44.7%)
Two	94 (55.6%)	371 (67.3%)	264 (55.3%)
**Vaccination card**			
One	28 (33.7%)	36 (20.9%)	81 (38.9%)
Two	55 (63.3%)	136 (79.1%)	127 (61.1%)
		**VE (95%CI)**	
**Vaccine efficacy**		39% (2 to 62)	8% (-77 to 52)

* Based on scar, matched and adjusted for year of birth, sex, known tuberculosis contact, water supply and income of the head of the family.

Another way at summarizing the same data is that the HU controls had roughly 1.75 odds of having received two BCGs than neighbourhood controls (95% 1.34–2.28). This will be explored further later.

Health Unit and neighbourhood controls were similar in relation to age and history of contact with TB. Health Unit controls had a slighter higher proportion of females. This was due not to refusals (which we estimate to have been fewer than 3%), but to the higher proportion of females among health centre users. Socio-economic status was measured through employment status and income of the head of the family; ownership of goods (washing machine, fridge and videocassette), and of whether the house had access to piped water in at least one of the rooms. Neighbourhood controls were worse off than Health Unit controls in most, but not all, socioeconomic indicators. They had statistically significantly higher levels of unemployment, and less ownership of a washing machine. Not statistically significant but also slightly worse among neighbourhood controls were income and ownership of videocassette. Ownership of a fridge was the same in the two control groups; and neighbourhood controls had statistically significantly more piped water in their households (Table 2).

**Table 2:** Distribution of biological features, contact with Tb and socio-economic characteristics in HU and neighbourhood controls

**Variables**	**Health Unit Control**		**Neighbourhood**		**P value**
			
	N°	%	N°	%	
**Sex**					0.063
Male	210	38.1	209	43.8	
Female	341	61.9	268	56.2	
**Tb contact**					0.520
Yes	72	13.1	56	11.7	
No	479	86.9	421	88.3	
**Head of family employed**					**0.026**
Yes	400	72.6	315	66.2	
No	151	27.4	161	33.8	
**Income of the head of family**					0.152
<1 MW*	31	5.63	33	6.92	
1 to <2MW	103	18.69	88	18.45	
2 to <5MW	45	8.17	30	6.29	
≥ 5MW	28	5.08	12	2.51	
No reported or not known	344	62.43	314	65.83	
**Piped water**					**<0.001**
Yes	503	91.5	467	98.5	
No	47	8.5	07	1.5	
**Washing machine**					**0.007**
Yes	134	24.3	81	17.3	
No	417	75.7	386	82.7	
**Frigidaire**					0.929
Yes	507	92.0	429	91.9	
No	44	8.0	38	8.1	
**Videocassette**					0.088
Yes	221	40.1	163	34.9	
No	330	59.9	304	65.1	

*MW: the income level of the head of the family was relative to the minimum wage (MW). MW<1 was the baseline. 1–1.9+ = US$100–US$199/month; 2–4.9 = US$200–US$499/month; 5+ = US$>=US$500/month.

The proportion vaccinated in the two control groups was different by age. This was more marked in the age group 7–9, where 56% of HU controls had two scars and only 19% of neighbourhood controls did. The proportions vaccinated in the other age groups were, in HU controls and in neighbourhood controls, for ages 10–14, 80% and 63.2% and for ages 15 and over, 62% and 57%.

Table 3 shows the OR of having a second BCG and the other variables separately for HU and neighbourhood controls. None are statistically significantly associated with a second dose BCG vaccine in either control group, except that in the neighbourhood control group the association between owning a video and having a second BCG dose is of borderline significance, and the associations between ownership of other goods, although not significant, are in the direction expected – with the wealthier having a higher coverage.

**Table 3:** Association between potential confounding variables and second dose BCG in neighbourhood and HU controls.

	**Neighbourhood controls**		**HU controls**	
	
**Variables**	**Two BCG/One BCG**		**Two BCG/One BCG**	
		
	OR	95% CI	OR	95% CI
**Sex**				
Male	1		1	
Female	1.17	0.81–1.68	0.82	0.57–1.19
**Contact with TB**				
Yes	1.18	0.67–2.08	1.12	0.65–1.91
No	1		1	
**Head of family employed**				
Yes	1.40	0.95–2.06	1.21	0.71–1.89
No	1		1	
**Income of the head of family**				
<1 MW*	1		1	
1 to <2MW	1.82	0.81–4.10	0.97	0.41–2.28
2 to <5MW	1.62	0.59–4.56	1.05	0.39–2.82
≥ 5MW	0.94	0.25–3.52	0.63	0.22–1.83
Not reported or not known	1.03	0.50–2.11	1.01	0.46–2.22
**Piped water**				
Yes	1.66	0.37–7.50	0.60	0.30–1.22
No	1		1	
**Washing machine**				
Yes	1.02	0.63–1.66	1.24	0.81–1.90
No	1		1	
**Frigidaire**				
Yes	1.57	0.80–3.06	0.76	0.38–1.51
No	1		1	
**Videocassette**				
Yes	1.42	1.01–2.19	0.91	0.63–1.30
No	1		1	

*MW: the income level of the head of the family was relative to the minimum wage (MW). MW<1 was the baseline. 1–1.9+ = US$100–US$199/month; 2–4.9 = US$200–US$499/month; 5+ = US$>=US$500/month.

It is clear that controlling for potential confounding variables did not change the finding of a higher vaccine coverage in Health Unit controls than in neighbourhood controls; in fact the OR is remarkably robust to the control of each of the variables (table 4).

**Table 4:** How OR of having a second BCG dose (in HU controls as compared to neighbourhood controls) varies when controlling for biological and social variables

**Adjustment variables**	**OR of having a second BCG given being a HU control compared to being a neighbourhood control**	
	
	OR	95%CI
Adjusted matched*	1.81	1.37–2.40
**Controlled for**		
Sex	1.83	1.38–2.42
Contact with TB	1.81	1.37–2.40
Head of family employed	1.79	1.35–2.38
Income of the head of family	1.83	1.38–2.43
Piped water	1.77	1.33–2.35
Ownership of		
Washing machine	1.84	1.36–2.45
Frigidaire	1.87	1.40–2.48
Videocassette	1.85	1.39–2.46

*Matched, controlling for year of birth as a continuous variable

## Discussion

The proportion who had received BCG revaccination was sufficiently different in Health Unit attenders controls and in neighbourhood controls to substantially bias the estimate of protection; the estimate using the neighbourhood control group was consistent with that from the RCT. This overestimation when using the Health Unit attenders group, remained after adjusting for social and biological variables and for contact with a case of TB. Although this could still be caused, to a certain degree, by unmeasured differences in the population, it is likely that the large part of this difference resulted from the fact that they were attendees of the Health Unit.

There is only one limitation of this study: neighbourhood controls, although born at the same time as HU controls were ascertained on average two years after Health Unit controls, and could have received vaccination in these two years or maybe the population changed over two years. We examined the card of neighbourhood controls that had a card, and none had the vaccine in the previous two years. To explore the degree of mobility in the study population we analyzed replies to two questions from the questionnaire. The proportion not born in Recife was about 10% in cases, in neighborhood controls and in HU controls. The proportion that moved to Recife in the previous 2 years was available only for cases and HU controls; this was under 1% in both groups. So it is clear that this is a remarkably stable population and changes in the population in the two years between recruitment of HU and neighborhood controls were unlikely to be responsible for the lower vaccine coverage in population controls.

Our results are similar to those of Heinemann *et al*[4] who found a higher frequency of exposure in hospital than in neighbourhood controls. They also coincide with those of Tell *et al*.(1991)[10] and Morabia *et *al. (1996)[5] in the sense that the frequency of exposure was influenced by the attendance of health facilities.

A novel aspect of our study is that we were able to adjust the OR of having a second BCG given being a HU controls compared to being a neighbourhood control for biological and social characteristics of the two control groups, to explore if these characteristics were behind the bias; we observed that controlling for none of the available variables changed the increased vaccination in Health Unit controls compared to population controls. To investigate questions related to socio-economic factors the questionnaire used a set of questions from the Brazilian demographic census [19] on characteristics of the individual, the head of the household and the household itself. These covered composition of the family, years of schooling, type of work, whether at work, income, characteristics of the household and ownership of goods. We do not mean here that each one has a specific effect on the risk of tuberculosis but that among them they capture enough aspects of complex social structure related to disease; and since controlling for those did not remove the bias, we are confident that the difference is not due to differences in socio-economic factors between the two control groups.

The Health Unit controls in our study were selected from attenders to the same Health Unit the cases had their health care from before being diagnosed with tuberculosis. The rationale for choosing controls from the same Health Unit was that they represent the population who would have become study cases had they developed the outcome of interest, and thus they would represent the source population of cases; in addition, logistically, Health Unit controls are easily identified and are more likely to be cooperative. We found that Health Unit attenders controls had a higher coverage of BCG revaccination than neighbourhood controls, therefore producing an overestimate of the protective effect of revaccination when compared to the RCT results [14]. The increased vaccine coverage in Health Unit attenders could be a result of their being registered: in this case registration in itself would indicate greater health awareness and willingness to be vaccinated. A more likely explanation is that the Health Units offer BCG vaccination to those who attend the health service for other reasons, and attenders have a higher rate of contact with the service in the past than those just registered there. Selecting Health Unit attenders controls lead to a distortion in the estimate of the protective effect of revaccination with BCG, as the frequency of revaccination in this group was greater than in neighbourhood controls and cases. Hospital or Health Unit based studies on the protective effect of vaccines, which disregard the role of these services in the delivery of vaccination, may be vulnerable to bias. In our study this point became evident only because a randomised control trial was conducted and a second group of controls – neighbourhood – was used. The best choice of controls is a random sample of the population from which the cases originated. Although our neighbourhood controls were ascertained 2 years later, we were able to show that this did not change their vaccine coverage, and thus are confident that they are a good representation that of the population that produced the cases.

## Conclusion

As the choice of the most adequate control group implies prior knowledge of selection probabilities which is hardly available, all efforts should be taken in the planning of the study to minimize selection bias; the use of two control groups seems to be a valuable tool when there is no confidence on which group is representative of the background rate of exposure in the source population of the cases, but particular care should be taken to avoid selecting controls as attenders of the institution that delivers the intervention under study.

## Competing interests

The author(s) declare that they have no competing interests.

## Authors' contributions

OD, RX and LR have made substantial contributions to conception and design, analysis and interpretation of the data, have been involved in drafting and revising the manuscript. MA, UM, WS and PV participated in the design of the study, acquisition of data, data analysis and interpretation and revision of the manuscript. All authors have given final approval of the version to be published.

## Pre-publication history

The pre-publication history for this paper can be accessed here:


